# Managing health sciences libraries in a time of change

**DOI:** 10.29173/jchla29839

**Published:** 2025-04-01

**Authors:** Melissa Caines

**Affiliations:** Library Services, Manager Canadian College of Naturopathic Medicine New Westminster, BC, and Toronto, ON, Canada

Joseph CB, Stephenson PL, editors. **Managing health sciences libraries in a time of change**. Lanham, MD: Rowman & Littlefield; 2024. Hardcover: 128 p. ISBN: 978-1-5381-7008-3. Price: USD$115.00. Available from: https://rowman.com/ISBN/9781538170083/Managing-Health-Sciences-Libraries-in-a-Time-of-Change.

The field of health sciences librarianship is no stranger to changes: from card catalogues, print collections, desk reference, and patiently waiting weeks or months for an inter-library loan to 24 hour access to electronic resources, virtual reference, and, quite often, same-day document delivery services. The internet, at first a disruptive technology, is now a ubiquitous part of daily life. *Managing health sciences libraries in a time of change* seeks to reflect on the role and identity of health sciences librarian leaders today and the skills they need to “adapt to change and remain flexible [...] while maintaining successful library operations” [1]. Specifically written with health sciences librarians interested or involved in library leadership in mind, this book is “designed to serve as a text for students and as a resource for practicing health sciences librarians” [1]. What results is a broad, yet comprehensive work exploring key concepts in health sciences library leadership, which complements Crum & Nuñez's (2023) publication, *Essential leadership skills for health sciences information professionals* [2]. Whereas *Managing health sciences libraries in a time of change* offers an insightful overview of the broader picture (combined with actionable strategies) for aspiring or current library leaders, *Essential leadership skills for health sciences information professionals* delves into specific areas such as project management and strategic planning, offering detailed guidance in those focused aspects. With 70 years of combined experience in health sciences librarianship and active participation in the Medical Library Association (MLA), editors Claire B. Joseph and Priscilla L. Stephenson provide the right expertise and dedication essential in bringing together this succinct primer, with chapters written by contributing authors (seven directors and one associate dean) from academic health sciences libraries and medical centres in the United States. Joseph is the Medical Library Director at Mount Sinai Nassau Hospital in Oceanside, New York and, before her retirement in 2022, Stephenson was Chief, Library Service for James A. Haley Veterans’ Hospital in Tampa, Florida. In addition to editing, Joseph and Stephenson are also contributing authors.

*Managing health sciences libraries in a time of change* is divided into 10 chapters, each focusing on a different concept, preceded by a preface providing context and setting the stage for the important discussions that follow. Chapters one and two give an overview of health sciences library leadership and trends in health sciences libraries, while chapter three offers an impressive breadth and depth of information on practical and approachable strategies to manage diversity, equity, inclusion, and belonging (DEIB). Chapters four through nine cover supporting staff, learning about leadership, management, mentoring, and accreditation standards (from a U.S. perspective). The final chapter, chapter 10, focuses on the unique dynamics of small health sciences libraries. Academic libraries, medical centre libraries, and smaller health sciences libraries are all given space in this book. Through interviews with health sciences leaders, chapter eight provides a clear roadmap for library professionals interested in health sciences library leadership. This chapter identifies four key objectives essential for embarking on this path: “breadth of experience,” “focused preparation,” “mentors,” and “recognizing/developing leadership potential” [1]. “Breadth of experience” refers to the various backgrounds and experiences many of us bring to our profession, regardless of whether this experience stems from the health sciences or other disciplines and careers. The remaining objectives emerge as themes throughout several other chapters, reflecting their roles in supporting health sciences library leadership. These objectives can be used to focus one’s personal leadership journey or can provide a much-needed lifeline for those “accidental health sciences librarian” [3] leaders finding themselves in a management role due to a vacancy or other serendipitous opportunity.

With the book’s reflection on change in today’s context, a few notable themes emerged across several chapters: the importance for health sciences libraries to align with their parent organization’s mission (chapters one, five, six, and nine), post-pandemic lessons from COVID-19 (chapters two through six), the 2020 murder of George Floyd highlighting systemic racism (chapters two, three, five, and six), and Fobazi Ettarh’s coining of the term “vocational awe” [3] (chapters three, four, and five). “Vocational awe describes the set of ideas, values, and assumptions librarians have about themselves and the profession that result in notions that libraries as institutions are inherently good, sacred notions, and therefore beyond critique” [4]. In her article, Ettarh demonstrates how “vocational awe directly correlates to [...] burnout and low salary,” [4] arguably critical considerations for library leaders striving to retain top talent while fostering a supportive and inclusive work environment prioritizing work-life balance. For readers interested in delving deeper into Ettarh’s concept of “vocational awe,” *Knowledge justice* offers invaluable perspectives by Indigenous, Black, and People of Colour (IBPOC) scholars through the theoretical perspective of critical race theory (CRT) [5].

Given the U.S. context of *Managing health sciences libraries in a time of change*, adjustments specific to the Canadian landscape need to be considered. For example, a notable omission from the book is the topic of decolonization and Truth and Reconciliation in libraries. Health sciences library leaders in Canada (and beyond) can look to the field of Indigenous librarianship alongside the work of Indigenous librarians, such as the recommendations in the *CFLAFCAB Truth and Reconciliation report* [6], and follow the ongoing work of the National Indigenous Knowledge and Language Alliance (NIKLA) [7]. A second omission is the lack of any specific mention of generative artificial intelligence (AI). Generative AI, however, fits into the book’s overall discussions around trends and changes in technology. Library professionals have long been accustomed to navigating significant changes and we will once again rise to the challenge, giving us more reason to engage in conversations around library advocacy and to demonstrate library value while aligning with and supporting the vision and mission of the parent institution.

In summary, the authors’ writing is clear and accessible, making the various concepts and themes easily digestible and actionable for readers. I particularly appreciated the chapter that resonated with my experiences working in a small library environment where I juggle managerial and leadership tasks. This chapter offered valuable insights and practical guidance that align closely with the multifaceted responsibilities of such roles. Each chapter is designed to stand alone, allowing librarians to engage independently with individual, yet integral, topics while still contributing to the book’s overall framework supporting health sciences library leaders. Together with end-of-chapter discussion questions, this book is an ideal addition to health sciences courses in library Master’s programs, as well as a resource for current and aspiring health sciences librarians. I highly recommend this book; not only for current or aspiring leaders, but for all health sciences library professionals as it offers broader insights into health sciences librarianship, advocacy ideas to engage with managers and leaders (particularly in the DEIB and staff support chapters), and helpful guidance for those who may transition into managerial or leadership roles in the future.
